# Clinical factors predicting ureteral stent failure in patients with external ureteral compression

**DOI:** 10.1515/med-2021-0345

**Published:** 2021-09-02

**Authors:** Kuan Ju Wu, Yi Zhong Chen, Marcelo Chen, Yu-Hsin Chen

**Affiliations:** Department of Urology, Mackay Memorial Hospital, Taipei, Taiwan; Department of Medicine, Mackay Medical College, New Taipei City, Taiwan; Institute of Pharmacology, National Yang Ming Chiao Tung University, Taipei, Taiwan

**Keywords:** external ureteral compression, double J, hydronephrosis, pyuria, prediction factors

## Abstract

Double-J stent (DJ) placement is usually the treatment of choice for relieving external compression of the ureter. However, in some cases DJ function may become impaired and a percutaneous nephrostomy (PCN) may be required. Previous studies have reported different predictive factors for choosing PCN or DJ insertion as the initial treatment. In this study, we analyzed the risk factors for DJ failure in patients with external ureteral compression. Our results showed that the patients with moderate and severe hydronephrosis (*p*-value = 0.0171 and 0.0249, respectively), preexisting pyuria (*p*-value = 0.0128), or lower ureter obstruction (*p*-value = 0.0305) were more prone to DJ laterality. Age was also an important predictor. Urologists should pay more attention to these patients and consider PCN as the initial treatment.

## 1 Introduction

External compression of the ureter can cause ureter obstruction and lead to hydronephrosis. Possible causes of external compression include retroperitoneal fibrosis or malignancies such as colon cancer, gynecologic (GYN) cancer, and urological cancer [1]. Hydronephrosis should be treated as soon as possible, since extended obstruction often causes symptoms such as infection, flank pain, and even renal function damage. Moreover, previous studies have shown that chronic kidney disease is linked to cardiovascular disease and an increased risk of mortality in the general population [2,3]. A two- to four-fold increase in cardiovascular disease has been reported in patients with impaired kidney function [2]. In addition, preoperative acute kidney injury has been shown to increase perioperative mortality and morbidity [4,5]. Therefore, preservation of renal function after postrenal obstruction is vital.

For urologists, double-J stent (DJ) insertion is often considered as the initial treatment for decompression. However, DJ function failure may occur in some patients, necessitating the use of other methods to relieve obstruction [6,7]. Furthermore, a previous study reported that DJs can malfunction in less than 1 year in patients with risk factors such as mild renal insufficiency at baseline and metastatic disease requiring chemotherapy or radiotherapy [8]. Many new materials and techniques have been developed to improve kidney function and relieve obstructions, such as tumor stents and segmental metal mesh stents [9]. These strengthened stents may be able to solve the problem of DJ dysfunction related to external compression. However, the results of previous studies with regards to other risk factors for DJ dysfunction have been inconsistent. Therefore, in this study, we reviewed data from Mackay Memorial Hospital to identify risk factors that may influence DJ’s durability.

## 2 Materials and methods

We retrospectively reviewed medical records from January 2011 to December 2018 of patients who received DJ insertion due to external compression. All the patients were treated with polymeric ureter stents (6.0 Fr and 22–26 cm long, Universa Firm and Soft Ureteral Stent; Cook^®^ Medical, Bloomington, IN). The interval between stent changes was 3 months. Patients who lost to follow-up and who died during the data collection period were excluded. Data including age, gender, type of malignancy, unilateral or bilateral DJ insertion, preoperative creatinine level, postoperative creatinine level, degree of hydronephrosis, obstruction level (defined as the upper, middle, or lower ureter according to the location above, over, or below the sacroiliac joint), and urine analysis were collected for further analysis. DJ insertion in both ureters of one patient was considered to be insertion in two ureteral units.

The degree of hydronephrosis was evaluated by kidney ultrasound before the DJ insertion. The grade of hydronephrosis (mild, moderate, and severe) was classified according to the Society of Fetal Ultrasound grading system. Pyuria was defined as the presence of more than six neutrophils per high power field of mid-stream urine before DJ insertion. DJ failure was considered when at least one of the following criteria was met: (1) hydronephrosis upgrade, (2) *a* > 150% increase in creatinine level from baseline, (3) percutaneous nephrostomy (PCN) placement if the ureteral stent could not be replaced or at the discretion of each attending urologist according to clinically significant symptoms, and (4) DJ dislodgement or malposition [10]. Area under the receiver operating characteristic curve (AUROC) analysis was used for continuous data including age and preoperative creatinine level. Other non-continuous factors were analyzed using univariate and multivariate analyses. We used logistic regression to analyze whether these factors could predict DJ function failure. Odds ratios (ORs) were also calculated to examine whether there was an increased risk of DJ failure. Kaplan–Meier survival curves were used to determine DJ durability.

This study complied with all relevant national regulations and institutional policies, and was approved by the Institutional Review Board of Mackay Memorial Hospital.

## 3 Results

Forty-seven patients in whom a total of 61 DJs were inserted were enrolled. The age of the patients ranged from 40 to 89 years. There were 3 males and 44 females, who received 3 and 58 DJs, respectively. The profile of the patients is shown in Table 1.

**Table 1:** Ureteral stent (DJ) profile

	No. of DJs (fail/not fail)
Total stent number	61 (31/30)
Age	
≤60 years	30 (12/18)
>60 years	31 (19/12)
Gender	
Male	3 (2/1)
Female	58 (29/29)
Cancer type	
GI	11 (6/5)
GYN	50 (25/25)
Cervical cancer	32 (19/13)
Ovarian cancer	6 (4/2)
Benign myoma	12 (2/10)
Hydronephrosis grade	
Mild	31 (10/21)
Moderate	21 (14/7)
Severe	9 (7/2)
Obstruction site	
Lower	37 (23/14)
Non-lower	24 (8/16)
Pyuria	
No	11 (1/10)
Yes	50 (30/20)
Unilateral or bilateral	
Unilateral	34 (15/19)
Bilateral	27 (16/11)
Laterality	
Left	31 (17/14)
Right	30 (14/16)

GI: gastrointestinal; GYN: gynecological.

The most common cause of compression was a GYN tumor (50 DJs for GYN tumors and 11 DJs for GI tumors) and most GYN tumors were cervical cancer. With regards to hydronephrosis, 31, 21, and 9 DJs were inserted in patients with mild, moderate, and severe hydronephrosis, respectively. The external compression level was at the lower ureter in approximately 60% of the ureteral units, with the other 40% being at the middle or upper ureter (which we combined into a non-lower group). Most of the patients (approximately 80%) had pyuria before DJ insertion. There was no significant difference in laterality.

**Figure 1:**
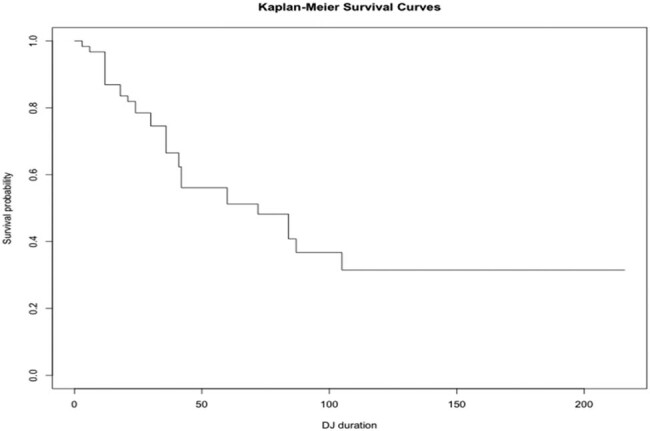
Kaplan–Meier survival curves. The average DJ stenting duration was 54.23 months.

As listed in Table 2, a total of 31 ureteral units failed. Of these 31 failures, 41.9% were due to *a* > 150% increase in creatinine level from baseline, 38.7% were caused by an inability to replace the ureteral stent or at the discretion of each attending urologist according to clinically significant symptoms, 12.9% were due to an upgrade in hydronephrosis, and 6.4% were due to DJ dislodgment or malposition. In the increased creatinine and upgraded degree of hydronephrosis groups, the patients were requested to keep ureteral stent insertion instead of performing PCN mainly due to restricted physical activity and negative impact on their quality of life. The average DJ stenting duration was 54.23 months as per the Kaplan–Meier survival curves shown in Figure 1.

**Table 2:** Causes of stent failure

	Number (%)
Total failure number	31
Change to PCN	12 (38.7%)
Increase in creatinine	13 (41.9%)
Hydronephrosis upgrade	4 (12.9%)
DJ dislodgment or malposition	2 (6.4%)

PCN: percutaneous nephrostomy.

Change to PCN: The ureteral stent could not be replaced or at the discretion of each attending urologist.

Univariate analysis revealed that there were significant differences in hydronephrosis grade, obstruction level, and preoperative pyuria between patients with or without DJ failure. Those who had a moderate and severe hydronephrosis grade had a higher risk of DJ failure compared to those with a mild grade (OR = 4.2 and 7.35 and *p*-value = 0.0171 and 0.0249, respectively). Obstruction level also influenced DJ function, and external compression at the lower ureter was associated with a higher risk of DJ laterality (OR = 0.30 and *p*-value = 0.0305). Preoperative pyuria status had a significant negative effect on DJ function (OR = 15 and *p*-value = 0.0128). Other parameters including age greater than 60 years, gender, preoperative creatinine level, cancer type, unilateral or bilateral, and lateralization were not significantly different (Table 3).

**Table 3:** Univariate analysis of DJ failure

	Odds ratio	*p* value
Age		
≤60 years		
>60 years	2.38	0.099
Preoperative Cr		
≤1.2 mg/dL		
>1.2 mg/dL	0.43	0.265
Cancer Type		
GYN		
GI	1.2	0.785
Hydronephrosis grade		
Mild		
Moderate	4.2	**0.0171**
Severe	7.35	**0.0249**
Obstruction site		
Lower		
Non-lower	0.30	**0.0305**
Pyuria	15	**0.0128**
Unilateral or bilateral		
Unilateral		
Bilateral	1.84	0.242
Lateralization		
Right		
Left	0.72	0.524

Bold values denote statistical significance at the *p* < 0.05 level.

Multivariate logistic regression analysis revealed that only severe hydronephrosis grade (OR = 34.89 and *p*-value = 0.0194) and preoperative pyuria (OR = 43.83 and *p*-value = 0.0153) were significant predictors of DJ failure (Table 4).

**Table 4:** Multivariate analysis of DJ failure

	Odds ratio	*p* value
Age		
≤60 years		
>60 years	0.77	0.7407
Preoperative Cr		
≤1.2 mg/dL		
>1.2 mg/dL	0.33	0.2579
Cancer type		
GYN		
GI	0.48	0.4961
Hydronephrosis grade		
Mild		
Moderate	4	0.074
Severe	34.89	**0.0194**
Obstruction site		
Lower		
Non-lower	0.28	0.1071
Pyuria	43.83	**0.0153**
Unilateral or bilateral		
Unilateral		
Bilateral	2.66	0.211

Bold values denote statistical significance at the *p* < 0.05 level.

**Figure 2:**
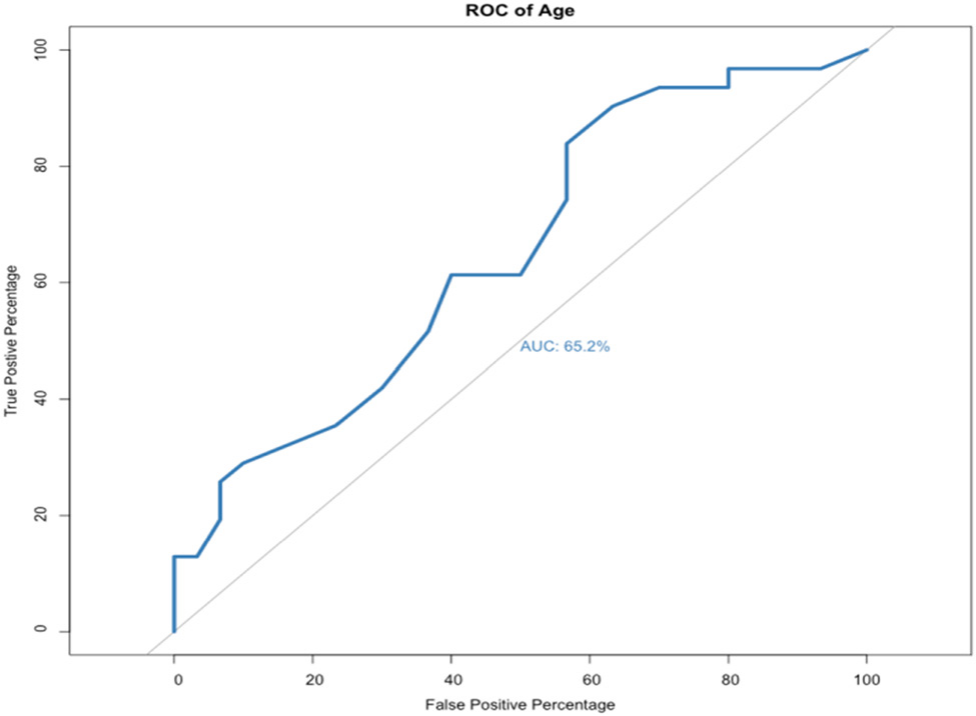
AUROC curve of age (OR = 1.08, *p*-value = 0.0214, specificity = 43.3%, and sensitivity = 83.9%).

AUROC analysis was used to analyze continuous data including age, preoperative creatinine level, and changes in creatinine level after 12 months of follow-up (Figure 2). The AUROC for age was 0.652 (OR = 1.08, *p*-value = 0.0214, specificity = 43.3%, and sensitivity = 83.9%). This indicated that age could be a predictor of DJ failure.

**Figure 3:**
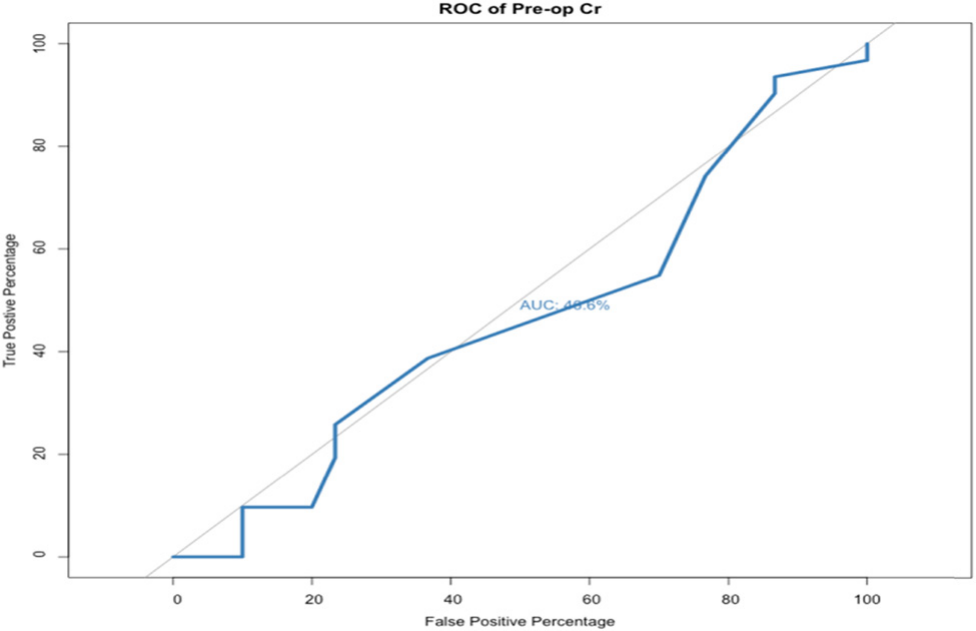
AUROC curve of preoperative creatinine (OR = 0.8, *p*-value = 0.287, specificity = 13.3%, and sensitivity = 93.5%).

The AUROC curve of preoperative creatinine level was 0.406 (OR = 0.8, *p*-value = 0.287, specificity = 13.3%, and sensitivity = 93.5%) indicating that preoperative creatinine level was not a good predictive factor (Figure 3).

The change in creatinine level after 12 months of follow-up is shown in Table 5. Creatinine level improved with 17 DJs (defined as the creatinine-negative group), of which 8 eventually failed and the other nine kept functioning. The positive group had no decrease in creatinine.

**Table 5:** Postop creatinine at 12 months

	No fail	Fail
Creatinine negative	9	8
Creatinine positive	17	22
NA	4	1

Creatinine = (Postop 12 months Cr – Preop Cr)/(Pre-op Cr).

Creatinine ≥0: Creatinine-positive, Creatinine <0: Creatinine-negative.

In the positive group, 22 DJs failed and 17 DJs still had proper function. Only 5 DJs did not have regular follow-up data (four of which remained functional).

**Figure 4:**
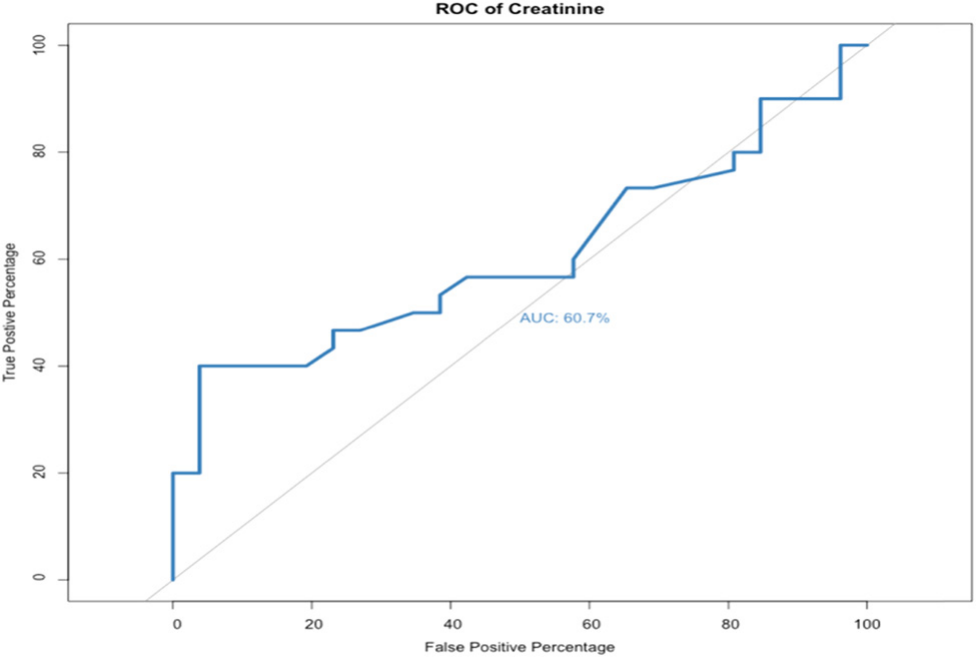
AUROC curve of change in creatinine level (OR = 2.62, *p*-value = 0.0576, specificity = 96.2%, and sensitivity = 40.0%).

The AUROC curve of changes in creatinine level at 12 months of follow-up was 0.607 (odds ratio = 2.62, *p*-value = 0.0576, specificity = 96.2, and sensitivity = 40.0). This indicated that the DJ failure rate increased with increasing postoperative creatinine level (Figure 4).

## 4 Discussion

In this study, a total of 61 DJs were regularly changed in 47 patients with ureteral external compression. The mean time of DJ function failure was 54.23 months. Previous studies have reported stent failure rates ranging from 16 to 53% [1,11,12,13,14,15,16], although these studies differed from each other in patient background, sample size, stent material, and types of malignancy. In our study, the stent failure rate was 50.8% (31/61), which is consistent with the previous studies. Several studies have reported different risk factors as predictors of stent failure, including preoperative serum creatinine [17,18,19,20], the degree of hydronephrosis [13,19,21], and GYN or GI cancer as the primary disease [1,6], and poor preoperative performance status [13,20]; however, the findings remain controversial. In addition, Pavlovic et al. described DJ failure as a prognostic factor for mortality and a sign of cancer progression in patients with malignant extrinsic ureteral obstruction [11].

In this study, univariate analysis showed that hydronephrosis grade, obstruction level, and preoperative pyuria were independent predictive factors for DJ failure. Other factors including age, gender, preoperative creatinine level, cancer type, unilateral or bilateral, and lateralization were not significantly associated with DJ failure. Hydronephrosis grade was significantly associated with DJ function failure, and more severe hydronephrosis was associated with a higher risk of DJ failure. The grade of hydronephrosis can be related to the severity of external compression, and this may have caused the DJ to fail. However, preoperative creatinine level was not associated with DJ failure. Miyazaki et al. found no connection between preoperative creatinine level and DJ failure, and concluded that preoperative creatinine may be influenced by contralateral renal function or the general condition of the patient, which are not linked to the patency rate of the DJ [22]. This result is in contrast to a study by Chow et al. who found that a high preoperative creatinine level was associated with DJ function failure [6]. The authors concluded that the link between high preoperative creatinine level and DJ failure may be inadequate urine production. In their study, the cutoff point of preoperative creatinine was 2 mg/dL [6]. The difference between our study and their study may be due to the different thresholds of preoperative creatinine used (2.0 and 1.2 mg/dL, respectively).

Preoperative pyuria was also a risk factor for DJ failure. Encrustation, colonization, and biofilm formation on the DJ can cause pyuria. Elevated levels of minerals in the urine or catalysis by urease-producing organisms may cause stent encrustation [23]. In addition, suture urolithiasis encrusted on nonabsorbable sutures in previous renal surgery (e.g., partial nephrectomy, pyeloplasty, and radical prostatectomy) may also result in pyuria [24]. All these factors could have led to DJ obstruction and caused DJ failure [10,25]. However, another study reported that bacteriuria rather than pyuria can cause this phenomenon [25]. Since urine cultures before DJ insertion are not routinely performed at Mackay Memorial Hospital, we could not analyze whether or not bacteriuria affected DJ function, which may be a limitation of the current study [1,25,26]. Previous studies have reported bacterial colonization in 42–90% of indwelling stents [25,27]. This is an important factor, and further studies are needed to clarify this issue.

Cancer type did not affect DJ function in our study. However, previous studies have reported that cancer type can be a prognostic factor, although with inconsistent findings [6,28]. Chow et al. reported that the time to DJ failure was longer in patients with lower GI cancer, which may suggest that the texture of lower GI cancer is softer than others [6,28]. However, Izumi et al. reported that GYN cancer was a significant favorable predictor of a longer duration to DJ failure [1]. However, they did not reach a conclusion as to why GYN cancer could be a favorable predictor.

Hung et al. [10] and Chow et al. [6] used 60 years old as the cutoff point to predict DJ failure, and showed statistical significance. Hung et al. showed no significant difference in age, however Chow et al. found that age may play a role in predicting DJ function failure [6,10]. As a result, we also used 60 years of age as the cutoff point for further analysis. Although univariate analysis did not show an association between age > 60 years and DJ failure, AUROC analysis showed that age was a good predictive factor for DJ failure. Hence, age may be a good predictive factor, but 60 years of age may not be an optimum cutoff value, and rather it should be treated as a continuous variable. Chow et al. concluded that older patients may have more comorbidities, which increases the risk of DJ failure [6].

Obstruction level was also a predictive factor for DJ failure in the present study, and the patients with lower ureteral obstruction due to external compression had a significantly higher risk of DJ failure compared to those with a non-lower obstruction level. However, this result differed from previous studies which reported ureter obstruction level was not a predictor for stent failure [1,21]. Yossepowitch et al. concluded that distal ureter obstruction was associated with a higher incidence of stent failure when considering intrinsic and extrinsic obstruction together, but no statistical significance was found when considering these two groups separately [21]. These different outcomes may be related to different inclusion criteria such as patient background, stent material, and etiology of the malignancies. Further large-scale studies are needed to confirm our findings.

In multivariate analysis, only severe hydronephrosis and preoperative pyuria were associated with DJ failure.

Ganatra and Loughlin reported that tumor invasion into the bladder played an important role in predicting stent failure [29]. Besides bladder invasion, the degree of hydronephrosis and preoperative performance status have been reported to be potential independent predictors of stent failure [16,30]. However, patients with tumor invasion into the bladder were not enrolled in this study.

There are some limitations to this study. First, this is a retrospective study and so selection bias may exist. Second, the indications for DJ insertion were not standardized and varied among urologists. Third, follow-up (duration, renal echography, and serum biochemistry) depended on the doctor’s judgment, and there was no standard duration. In addition, most of our patients were female with GYN cancers. We also found that age was a good predictive factor; however, the optimal cutoff point of age was inconclusive due to the small number of patients. Previous studies have discussed the effect of pre-stenting radiotherapy; however, this was difficult to trace on the charts we reviewed [31,32]. Previous studies have compared DJs made from different materials. For example, Hung et al. and Chow et al. concluded that metallic ureteral stents had superior extrinsic compression resistance and longer median durability compared to polymeric DJs [10,33,34,35,36]. However, we only included polymeric DJs and excluded metallic DJs. Therefore, we may have eliminated bias between different materials, which may have increased the ability to identify predictive factors of DJ failure [34,35,37]. Another limitation is that we only targeted patients who underwent successful stent placement initially, and excluded those with unsuccessful insertion. Finally, the number of cases was small, which made it relatively difficult to analyze all possible predictive factors.

In conclusion, despite the small sample size in this study, patients with moderate to severe hydronephrosis, preexisting pyuria, or lower ureter obstructions were more prone to DJ failure. In addition, age should be taken into account as a predictive factor. Since DJ decompression for ureteral external compression is the most common initial treatment, clinicians should pay more attention to patients with these risk factors. In addition, shortening the follow-up period and using PCN as the initial treatment can be considered.
